# Novel insights into the regulatory role of N6-methyladenosine methylation modified autophagy in sepsis

**DOI:** 10.18632/aging.205312

**Published:** 2023-12-18

**Authors:** Cheng-Fei Bi, Jia Liu, Xiao-Dong Hu, Li-Shan Yang, Jun-Fei Zhang

**Affiliations:** 1 Department of Emergency Medical, General Hospital of Ningxia Medical University, Yinchuan 750000, Ningxia, China; 2 Medical Experimental Center, General Hospital of Ningxia Medical University, Yinchuan 750000, Ningxia, China; 3 School of Clinical Medicine, Ningxia Medical University, Yinchuan 750000, Ningxia, China

**Keywords:** m^6^A methylation, autophagy, sepsis

## Abstract

Sepsis is defined as a life-threatening organ dysfunction caused by a dysregulated host response to infection. It is characterized by high morbidity and mortality and one of the major diseases that seriously hang over global human health. Autophagy is a crucial regulator in the complicated pathophysiological processes of sepsis. The activation of autophagy is known to be of great significance for protecting sepsis induced organ dysfunction. Recent research has demonstrated that N6-methyladenosine (m^6^A) methylation is a well-known post-transcriptional RNA modification that controls epigenetic and gene expression as well as a number of biological processes in sepsis. In addition, m^6^A affects the stability, export, splicing and translation of transcripts involved in the autophagic process. Although it has been suggested that m^6^A methylation regulates the biological metabolic processes of autophagy and is more frequently seen in the progression of sepsis pathogenesis, the underlying molecular mechanisms of m^6^A-modified autophagy in sepsis have not been thoroughly elucidated. The present article fills this gap by providing an epigenetic review of the processes of m^6^A-modified autophagy in sepsis and its potential role in the development of novel therapeutics.

## INTRODUCTION

Sepsis is a potentially fatal organ failure brought on by an improperly controlled host response to infection [1], with a high morbidity and mortality rate worldwide. In 2017, the World Health Assembly listed sepsis as a global health priority [2]. Sepsis is one of the leading causes of death in the intensive care unit (ICU) [3]. According to statistics, sepsis accounts for 20% of annual deaths worldwide [4, 5]. Sepsis has a very complicated etiology that involves pathophysiological processes such as an excessive inflammatory response, pyroptosis, immunological dysfunction, mitochondrial damage, coagulation failure, oxidative stress, apoptosis, and autophagy, ultimately leading to organ dysfunction [6–10]. Over the past few decades, there have been efforts to come up with sepsis treatment strategies. In recent years, much progress has been achieved in the anti-infective, fluid resuscitation, hemodynamic sustain and organ function support therapy of sepsis using microbiology facilities and nanotechnology drug delivery platforms [11, 12]. However, the current clinical management of septic patients is still supportive rather than curative. It must be acknowledged that sepsis is a challenging issue for ICU physicians to overcome due to its multi-causal nature. Therefore, it is essential to study the molecular mechanisms underlying the biological processes involved in sepsis in order to optimize treatment options for the condition.

To date, there are more than 100 recognized modifications involved in regulating the bio-metabolic processes of RNA [13]. The most well studied RNA modification to date is N6-methyladenosine (m^6^A) methylation. Human messenger RNAs (mRNA), ribosomal RNAs (rRNA), and small nuclear RNAs (snRNA) all carry the m^6^A modification. m^6^A methylation is a reversible posttranscriptional modification of mRNA and regulates mRNA biogenesis and function [14]. Such modification regulates multiple steps of RNA processing including splicing, export, localization, decay and translation. More than 12,000 m^6^A loci were found in more than 7,000 human gene transcripts using antibody-mediated capture and massively parallel sequencing-based m^6^A-seq [15]. Studies have shown that m^6^A modification-related proteins are strongly associated with disease severity and prognosis [16, 17]. Especially, m^6^A methylation plays an essential role in inflammation by regulating three inflammatory signaling pathways including MAPK, JAK/STAT3, and PI3K [18]. Further data have demonstrated that METTL14-mediated m^6^A methylation negatively regulates inflammatory response in the context of sepsis [19]. It was discovered that insulin-like growth factor 2 mRNA binding proteins (IGFBPs), one of the m^6^A methylation binding proteins, influence the initial stages of septic shock [20]. Moreover, entire or RNA-specific therapeutic treatment of m^6^A methylation dynamics may be helpful to prevent and mitigate sepsis-induced disseminated intravascular coagulation [21]. In general, growing data suggests that m^6^A methylation is a mechanism that affects the onset and progression of sepsis. However, the regulatory function and underlying mechanisms of m^6^A in sepsis haven’t been thoroughly illuminated.

Under physiological environments, autophagic activity is often limited and serves as the cell’s guardian. However, when cells are exposed to outside stimuli such as pathogenic bacteria, hypoxia, and endotoxins, autophagic activity is significantly increased. Clearly, defects in the selective regulation of autophagy may lead to disease [22]. Autophagy is activated in the early stages of sepsis, followed by a phase of impaired autophagy [23]. Previous researches have shown that autophagy activation during sepsis is crucial for preventing subsequent lung, renal, and cardiac injury [24–26]. Existing research recognizes the critical role played by m^6^A methylation modification-related proteins in the biogenesis of autophagy [27, 28]. There is a growing awareness about the biological significance of the m^6^A modification on the transcription and translation of genes related to autophagy as well as the overall impact of conferring RNA specificity [29].

**Figure 1:**
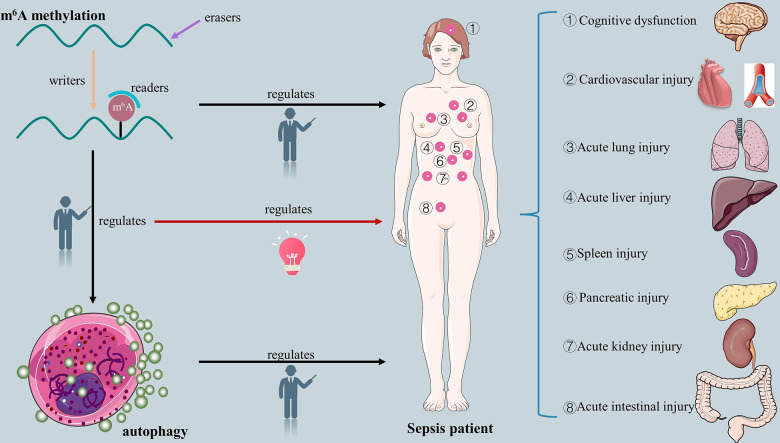
The overview of relationship between m^6^A methylation, autophagy, m^6^A-modified autophagy and sepsis.

Clinically, m^6^A methylation-modified autophagy mechanisms play a crucial role in improving the prognosis of patients with sepsis [30]. There is no doubt that the connection between m^6^A methylation and autophagy will offer fresh perspectives on the management of sepsis. However, the effect of m^6^A-modified autophagy in the pathophysiology of sepsis remains largely unclear. Therefore, it is essential to clarify the potential mechanisms that revealing the exact biological processes and specific organ function-protective roles of m^6^A-modified autophagy in sepsis. The relative evidence that supports whether m^6^A methylation modified autophagy influences the pathophysiological mechanisms of sepsis is compiled in this review. The graphical abstract of this study is shown in Figure 1.

## m^6^A methylation

### What is m^6^A methylation?

m^6^A methylation dynamically regulates RNA splicing, transport, localization, stability and translation [31]. m^6^A methylation, one of the common base modifications of mRNA, predominantly distributed in near stop codons, in 3’ UTRs [32] and within unusually long internal exons [15]. The blocked 5’terminal structure of heterogeneous nuclear RNAs, which exhibits striking similarities to one of the two varieties of blocked 5’ sequences seen in mRNAs, was discovered to be the site of m^6^A methylation as early as 1975 [33, 34]. Then, it has been discovered that the 5′ UTR’s m^6^A functions to stimulate mRNA translation when cells are under stress by taking the place of the 5’cap (which is the first step of most mRNAs translation) [35]. Of note, m^6^A is primarily found within the highly conserved consensus motif known as RRACH (R=G or A, H=A, C or U) in the majority of RNAs [36]. And then, m^6^A methylation also presences in a sequence context of UAC-(m^6^A)-GAGAA on top of a hairpin structure in transcript methionine adenosyltransferase 2A (MAT2A), which is mediated by methyltransferase-like 16 (METTL16) [37]. It is obvious that m^6^A methylation is an adenosine methylation at position N6, namely methylation of the sixth nitrogen atom on the RNA molecule adenosine. S-adenosylmethionine (SAM) provides nearly all the methyl groups necessary for cellular methylation reactions. The splicing of the MAT2A retained intron maintains high levels of intracellular SAM [38]. The specific mechanisms are as follows: the restriction of SAM prevents METTL16 from effectively inducing m^6^A methylation, which lengthens the time that it spends on a conserved hairpin (hp1) of MAT2A and promotes the splicing of retention intron, further provides enough SAM for m^6^A methylation.

### Who was involved in the m^6^A methylation?

There are three crucial m^6^A methylation modification-related proteins exist in the process of m^6^A methylation: m^6^A methyltransferases (writers), m^6^A demethylases (erasers) and m^6^A recognition factors (readers) [39]. According to recent studies, writers include methyltransferase-like 3 (METTL3), methyltransferase-like 14 (METTL14), methyltransferase-like 5 (METTL5), METTL16, Wilms tumor 1-associated protein(WTAP), Vir-like m^6^A methyltransferase associated (VIRMA), RNA binding motif protein 15 (RBM15) and zinc-finger CCHC domain-containing protein 4 (ZCCHC4), erasers include fat mass and obesity-related proteins (FTO) and alkB homolog 5 (ALKBH5), readers include the YTH structural domain family (YTHDF) 1-3, YTH structural domain containing family (YTHDC) 1-2, eukaryotic translation initiation factor 3 subunit A(eIF3), insulin-like growth factor 2 mRNA binding protein 1/2/3(IGF2BP1/2/3), heterogeneous nuclear ribonucleoprotein A2/B1(HNRNPA2/B1) and HNRNPG, HNRNPC [40–42]. The potential role of m^6^A methylation modification-related proteins on RNA metabolism and the outcome of disease are summarized in Supplementary Table 1 [43–79].

## How do m^6^A methylation modification-related proteins function during m^6^A methylation?

The METTL3-METTL14 compound is the ultimate important component in the writer proteins of m^6^A methylation. It is well established that the incidence of m^6^A methylation modification of various RNAs in mammals is inextricably linked to the activation of METTL3 and METTL14 [80]. In the process of m^6^A modification, METTL3 primarily functions as a catalytic core, while METTL14 provides a binding platform for RNA [81]. A recent study has shown that SUMOylation of METTL3 inhibits its m^6^A methyltransferase activity on RNAs [82]. The m^6^A methyltransferase’s core subunit, METTL14, works in stable heterodimer with METTL3 to catalyze m^6^A modification [83]. Subsequent research revealed that the methyltransferase activity of METTL3-METTL14 could only be elicited by the solution structure of the METTL3 zinc finger domain and not by the structural field of heterodimer between METTL14 and METTL3 [84]. Moreover, the METTL3-METTL14 complex is enlisted by WTAP, a regulatory subunit of the m^6^A methyltransferase complex, into nuclear patches that are enriched in pre-mRNA processing factors [85]. Prior works have also demonstrated that the mTORC1 modulates m^6^A methylation through regulating WTAP level to trigger the translational machinery for cell growth and proliferation [57, 86]. Contrary to the METTL3-METTL14 heterodimer, METTL16 is a single-component enzyme whose disordered loop is required to catalyze m^6^A methylation and whose N-terminal module is necessary for RNA binding [87]. Clearly, METTL16 in the cytoplasm and the nucleus serve different biological purposes. Only MAT2A mRNA and U6 snRNA were previously reported to directly deposit m^6^A from METTL16 [38, 88]. Reduced MAT2A mRNA degradation results from METTL16 localizing to hairpin 1 (hp1) on the 3’UTR of MAT2A mRNA and inducing MAT2A retained intron splicing [88]. In recent years, more RNAs with METTL16-mediated m^6^A methylation were reported in Supplementary Table 1 [43–79]. Additionally, wang, Fei et al. found that METTL16 facilitates mRNA 5 ‘cap-eIF4E recognition by sequestering eIF4E2 (translation initiation factor) [89]. Further studies suggest that this process of METTL16-mediated protein translation is independent of methyltransferase activity [89]. Therefore, in addition to catalyzing m^6^A methylation in the nucleus, METTL16 also participates in protein translation in the cytoplasm. The catalytic subunit m^6^A-METTL complex (MAC) and the regulatory subunit m^6^A-METTL-associated complex (MACOM) make up the m^6^A “writer”. The primary mechanism by which MACOM attaches to MAC is an interaction between WTAP and METTL3. Strikingly, WTAP and VIRMA comprise the basic structure of MACOM [90]. VIRMA recruits the METTL3/METTL14/WTAP, which are catalytic core components, to guide m^6^A methylation in 3’UTR and near stop codon of mRNA [91]. RBM15 plays a similar role to VIRMA in the methylation of m^6^A. RBM15 mediates m^6^A modification of targeted RNAs by targeting METTL3-METTL14 heterodimers to sites with or adjacent to m^6^A sites across the transcriptome [92]. ZCCHC4, a novel m^6^A methyltransferase that catalyzes m^6^A modification on rRNAs via binding to S-adenosyl-L-homocysteine, was recently reported [93]. ZCCHC4 is localized to the nucleolus, and ZCCHC4-mediated rRNA modification may also involve mRNA translation [94]. Further research established that ZCCHC4’s unique structural and enzymatic properties—namely, the formation of a complete RNA-binding surface by the association of the methyltransferase structural domain with the N-terminal GRF-type and C2H2 zinc finger structural domains and the C-terminal CCHC structural domain—are responsible for of its catalytic effect on rRNAs m^6^A modification [95].

m^6^A erasers predominantly catalyze demethylation of m^6^A-containing RNA. Two m^6^A demethylases have received a lot of attention to date: FTO and ALKBH5. FTO, one m^6^A eraser, primarily regulates the m^6^A modification in the nucleoplasm. FTO regulates pre-mRNA processing via its demethylation activity, which also influences mRNA stability close to the 7-methylguanosine cap, promotes cap-independent translation initiation at the 5’UTR, encourages exon jumping and alternative splicing at the pre-mRNA body, and modulates alternative poly(A) sites (APA) usage and 3’UTR length at the 3’UTR [96]. For instance, FTO can demethylate GAP-43 mRNA, and demethylation of GAP-43 mRNA may promote axonal elongation and regulate neural development [97]. ALKBH5, the other m^6^A eraser, localizes to nuclear speckles that are in charge of assembling mRNA processing factors. ALKBH5, a 2-oxoglutarate (2OG) and ferrous iron-dependent nucleic acid oxygenase (NAOX), has the potential to specifically bind single-stranded RNA attributed to a large loop (βIV–V) region that resembles the L1 loop of FTO [98]. ALKBH5’s demethylation activity has a momentous impact on gene expression, metabolism, and export of nuclear RNA (mainly mRNA), which regulates the biogenesis of m^6^A methylation on RNA [99]. In recent years, the crucial role of de-methylating of ALKBH5 in improving RNA stability has been particularly emphasized [100]. The demethylation of m^6^A modifications, exhibited by ALKBH5, improves the stability and expression levels of downstream RNAs that modulate heart regeneration and tumorigenicity [101, 102]. Does the m^6^A eraser’s demethylation activity work on methylated RNAs, though? A study offers an explanation: ALKBH5 and FTO keep their regulatory sites in an unmethylated stable state rather than reversing the methylated RNAs [103].

The variety of structural domains that m^6^A binding proteins possess allow for the division of these proteins into different families. Here, we’ll start out by introducing YTHDFs, which contain the YTH structural domain. YTH domain is known to directly bind the m^6^A base of methylated RNA [104]. YTHDF1 primarily recognizes the m6A methylation site of the downstream mRNA at the 3’UTR [66]. Mechanically, YTHDF1 recruits the transcripts of m^6^A-modified RNAs to facilitate their translation initiation [105], YTHDF2 induces the degradation of m^6^A-modified RNAs to decrease their stability [106], and YTHDF3 regulates the m^6^A methylation of downstream signal RNAs in synergy with YTHDF1 or YTHDF2 [107]. Contrary to these conventional beliefs, studies have proposed a novel unified model of m^6^A function, in which all m^6^A bits combine all three DF paralogs (YTHDF1, YTHDF2 and YTHDF3) in a fundamentally similar manner, influencing the degradation of mRNA through the interaction of these three key redundant DF proteins [108]. They did not, however, turn up any evidence that would support their hypothesis that these three DF proteins cooperating together have a role in promoting mRNA translation. Second, we’ll introduce the overview of YTHHDCs in the present paper, which share the same YTH structural domain. m^6^A methylation modification is a significant modality of regulation in mRNA splicing. The fate of the transcripts in terms of splicing dynamics and alternative splicing may be determined by early m^6^A sedimentation [109]. According to biochemical, structural, and transcriptome-wide PAR-CLIP (photoactivated ribonucleic acid enhanced cross-linking and immunoprecipitation) investigations, YTHDC1 is a nuclear RNA-binding protein that is responsible for recruiting mRNA splicing factors for pre-mRNA. The result from the current study has demonstrated that YTHDC1 promotes SRSF3 but antagonizes SRSF10 binding to RNAs at the m^6^A methylation site, further triggering the initiation of mRNA splicing [110]. It has been proposed that YTHDC2 may interact with translation and decay mechanisms in the context of particular binding to m^6^A in order to boost translation effectiveness and reduce the mRNA abundance of its targets [111]. Third, the identification of m^6^A by IGF2BPs depends on the K homology (KH) structural domain. Such m^6^A reading proteins selectively bind m^6^A-containing RNA using the KH structural domain and its flanking regions [104]. IGF2BPs have been reported to support the stability, storage and translation of their target mRNA transcripts by identifying their consensus GG-(m6A)-C sequences [78, 112]. The selectivity with which the remaining m^6^A reading proteins activate m^6^A causes them to be clustered together. Such m^6^A reading proteins bind m^6^A-containing transcripts through a m^6^A switch mechanism because the m^6^A modification weakens Watson-Crick base pairing of RNA and makes it easier for m^6^A reading proteins to recognize single-stranded RNA motifs; simultaneously, hnRNPA2B1 can also bind m6A-containing RNA with specificity by using the RRM structural domain and its flanking regions [104]. HNRNPG, a novel m^6^A methylation binding protein, binds purine-rich regions exposed by m^6^A modified RNA using its low-complexity region, regulating gene expression and selective splicing [42]. eIF3 binds directly to the 5’UTR m^6^A site of mRNA in the cytoplasm, which is sufficient to recruit the 43S complex and initiate translation in the absence of the cap-binding factor eIF4E [35, 113].

**Figure 2:**
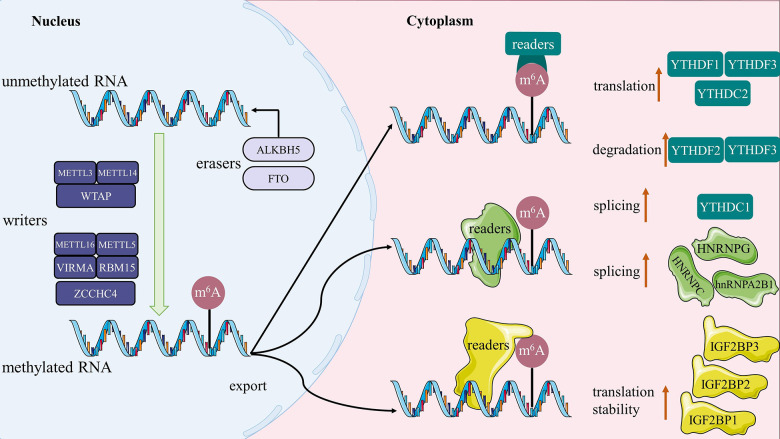
**The sketch map of m^6^A methylation.** Writers catalyze the m^6^A modification of RNA, erasers maintain the RNA in an unmethylated state, and the readers are ultimately responsible for determining the fate of the RNA (such as splicing, stability, degradation and translation).

It is still controversial what function m^6^A-related proteins have in the pathogenic and physiological processes of the disease. For example, analysis reports of TCGA data indicate that high expression of METTL3 is associated with unfavorable prognosis in CRC patients [114]. Conversely, clinical research has demonstrated that METTL14 deletion is related to a poor prognosis in patients with CRC [52]. By modification of m^6^A, METTL3 can also assist in regulating the cardiac homeostasis and hypertrophy [115]. WTAP was also identified as an independent predictor of prognosis for patients with hepatocellular carcinoma [58]. According to one study, testicular Leydig cells experienced an increase in m^6^A methylation modification of RNA due to the inhibition of FTO, which led to apoptosis [116]. By examining the expression of m^6^A-related regulators and the probability of overall survival in HNSCC, Yu, Dan et al. discovered IGF2BP2 to be an independent prognostic factor in patients [77]. To sum up, various regulations of m^6^A methylation modification-related proteins play a critical role in the corresponding modified RNAs’ transcription by affecting their splicing, export, translation, and stability, which ultimately influences the development of these modified RNAs-mediated diseases. The diagrammatic sketch of these regulatory mechanisms is shown in Figure 2. It follows that m^6^A methylation is expected to develop as a therapeutic target for human diseases.

However, as the field of study developed, we discovered that numerous m^6^A modification-related proteins interact to regulate the m^6^A methylation of targeted RNAs, rather than a single m^6^A modification-related protein, in the disease process. Many m^6^A methylation sites on SPRED2 mRNA have been found to be lost as a result of METTL3 deletion [117]. This impairs YTHDF1-mediated translation of the modified SPRED2 mRNA and increases NF-kB and STAT3 activation through the ERK pathway, which promotes tumor development and metastasis [117]. The level of SRY (sex determining region Y)-box 2 (SOX2) transcripts’ m^6^A methylation elevated as a result of METTL3. IGF2BP2 subsequently recognized methylated SOX2, maintaining its mRNA stability and expression. Ultimately, CRC development was triggered by high SOX2 expression [114]. However, when abundant in cellulose, METTL3’s role changes from catalyzing m^6^A methylation to promoting the initiation of mRNA translation. The production of dense polynucleotides, accelerated translation, and carcinogenic transformations all depend on METTL3-eIF3h interactions [118]. Moreover, another role m^6^A methylation frequently plays in disease development is targeted RNA degradation that is dependent on the METTL3-YTHDF2 interaction [73, 119]. METTL3 directs m^6^A modification of PKC-η, FAT4, and PDGFRA mRNAs to induce mRNA degradation via YTHDF2-dependent pathway, which promotes diabetes-related peripapillary cell dysfunction and stimulates retinal vascular complications [43]. As a result, METTL3 induces high levels of m^6^A modification in mRNA, and YTHDF2 identifies m^6^A sites in mRNA and promotes its degradation [120]. In the last resort, the synergistic effect of METTL3-YTHDF2 regulates the development of diseases via influencing disease-related genes expression. In a METTL3-FTO-dependent manner, m^6^A methylation plays the crucial role in the clinical and physiological processes of obesity cardiomyopathy, too [121]. ALKBH5 deletion leads to elevated m^6^A levels in downstream RNAs, and IGF2BP1 recognizes the exposed m^6^A sites and enhances their stability, thereby enhancing downstream RNA expression [122]. Similarly, ALKBH5-mediated m^6^A modification of its downstream targets is recognized by another m^6^A reader, YTHDF2, which is also responsible for degrading ALKBH5’s methylated downstream targets [123, 124]. Supplementary Table 1 [43–79] provides additional information on the essential role of writer/eraser-reader-dependent m^6^A methylation in the regulation of disease. In a word, m^6^A methylation is a dynamic and programmed process of RNAs modification. Even though each of the m^6^A modification-related proteins has a specific function, m^6^A modification of RNAs seems impossible to happen without the synergistic effect of these proteins. The level of RNAs’ m^6^A methylation in the nucleus is regulated by the m^6^A methyltransferases and demethylases, and the modified RNA enters the cytoplasm. Further affecting RNA splicing, degradation, stabilization, and translation are m^6^A binding proteins, which find and bind m^6^A residues on the transcript of the modified RNA. Therefore, this distinctive “writer/eraser-reader-dependent” paradigm for m^6^A methylation should be considered in the pathophysiological processes of disease, providing additional possibilities for therapeutic intervention.

Of course, the upstream signaling of m^6^A methylation-related proteins also influences their expression level, which in turn influences the level of downstream signaling that m^6^A regulates and, ultimately, the progression of the disease. For instance, Piwi-interacting RNA (piRNA)-14633 interacts with the 3’UTR of METTL14 to enhance the stability of METTL14 mRNA and encouraged the methylase activity of METTL14, promoting the m^6^A methylation levels of the downstream target (CYP1B1), and subsequently promoting the expression of CYP1B1, which in turn contributed to the oncogenesis of cervical carcinoma [53]. Additionally, by interacting with METTL3 and inhibiting its RNA methylation activity, cardiac-hypertrophy-associated piRNA (CHAPIR) prevents the m^6^A modification of PARP10 mRNA. This causes a blockage of the YTHDF2-mediated degradation of the PARP10 mRNA transcripts and an increase in PARP10 expression, which leads to cardiac hypertrophy [119].

## The regulatory role of m^6^A methylation in sepsis

A complicated systemic inflammatory response, immunological dysfunction, aberrant coagulation, oxidative stress, apoptosis, dysregulation of autophagy, and tissue damage are all factors in the pathogenesis of sepsis. m^6^A methylation facilitates these biological processes by differentially regulating specific RNAs. Trials have demonstrated a substantial correlation between m^6^A regulators including ALKBH5, HNRNPC, KIAA1429, WTAP, and YTHDF2 and 28-day cumulative mortality in sepsis patients. Of note, HNRNPC, KIAA1429, and YTHDF2 are protective genes with a hazard ratio (HR) < 1, but ALKBH5 and WTAP are dangerous genes with a HR > 1 [30]. Further research also confirmed the protective effects of FTO, HNRNPC, YTHDC1, and RBM15B in sepsis patients [125]. In one animal study, increased expression of METTL3 and low expression of METTL14, ALKBH5, FTO, and YTHDF2 were found following lipopolysaccharide (LPS) induction. Subsequent research demonstrated that m^6^A modification plays a role in the pathophysiology of sepsis and mediates sepsis-induced liver injury [126]. The m^6^A modification may have an intimate and intricate interaction relationship with the cardiovascular injury generated by the different physio-pathological conditions of sepsis. Shen et al. observed that downregulation of METTL3 and WTAP was partially responsible for the decrease in major m^6^A levels in aortic RNA during sepsis [21]. Additionally, there was a significant decrease in the levels of m^6^A modification in septic cardiac tissue, indicating a critical role for m^6^A modification in the pathogenesis of sepsis-related myocardial damage [127]. In summary, the evidence that is now available generally points to the possibility that therapeutic adjustments of cellular m^6^A methylation may assist with alleviating secondary organ dysfunction during sepsis.

We can’t only look at the overall degree of change in m^6^A methylation in sepsis, though, given the complicated pathophysiological mechanisms of sepsis and the dynamic and multifactorial role of m^6^A methylation. As a result, it is appropriate to incorporate the “reader/eraser-reader” model of m^6^A modification into the mechanistic investigation of sepsis and to clarify the specific molecular mechanisms of sepsis in the context of the dynamic process of cellular m^6^A modification. Data mining revealed that the majority of m^6^A-RNA methylation regulators’ expression was down-regulated in sepsis, with only a few up-regulated [128]. Recently, several *in vitro* experiments with the sepsis model observed abundant m^6^A methylation in LPS-induced cardiomyocytes (H9C2). Mechanically, METTL3 catalyzed m^6^A modification of HDAC4 mRNA, and IGF2BP1 identified the m^6^A site on HDAC4 mRNA and strengthened its stability, which consequently stimulates the inflammatory damage of cardiomyocytes induced by sepsis [129]. Obviously, METTL3-mediated m^6^A modifications on transcripts of numerous inflammatory signaling pathways are responsible for the excessive inflammatory responses and pyroptosis [130, 131]. More specifically, endotoxin invasion stimulates m^6^A methylation of intracellular inflammatory factors IL-6 and TNF-α transcripts in response to myocardial inflammation in sepsis [132, 133]. Likewise, YTHDF2 recognizes METTL3-mediated m^6^A modification of SLC7A11 mRNA and promotes the degradation of SLC7A11 mRNA, ultimately leading to ferroptosis in sepsis-induced myocardial injury [134]. A recent study reported that METTL3-induced m^6^A modification on ferroptosis was involved in the pathogenesis of sepsis-associated acute lung injury [135]. Conversely, there is a decreased m^6^A level in sepsis-induced acute respiratory distress syndrome (ARDS) *in vivo* and *in vitro*. Functionally, YTHDF1 recognized and stabilized METTL3-mediated m^6^A-modified tripartite motif-containing (Trim59) mRNA to protect the vascular endothelium against barrier dysfunction and inflammatory responses, which inhibits the evolution of ARDS during sepsis [136]. FoxO1/NF-κB is a recognized inflammatory signaling pathway that mediates the inflammatory response by promoting the generation of the inflammasome NLRP3. Previous studies have demonstrated that inhibition of FTO mediates m^6^A modification of FoxO1 mRNA and reduces its expression, thereby suppressing the inflammatory response in septic shock [137]. Moreover, inflammatory signaling pathway TLR4/NF-κB is negatively regulated by SOCS1 and Spi2a. It is understood that SOCS1 and Spi2a mRNA stability as well as translation are improved by METTL14-YTHDF1-dependent m^6^A methylation to prevent the progression of sepsis [19, 138]. Therefore, “writer/eraser-reader-dependent” m^6^A methylation may be a regulator of sepsis progression. It is concluded that the alterations in m^6^A modification during sepsis are closely associated with ferroptosis, pyroptosis, inflammatory and immune responses. Meanwhile, autophagy as a protective mechanism in sepsis and more m^6^A methylation regulating its biological role in the pathophysiological processes of sepsis need to be elucidated.

## The regulatory role of m^6^A methylation in autophagy

The regulatory role of m^6^A methylation in the development of autophagy must be established in order to gather evidence for the association between m^6^A methylation-modified autophagy and sepsis, which indicates that this RNA modification contributes essentially to the biological processes of autophagy initiation, extension, and maturation. Basal autophagy is tightly regulated by transcriptional and epigenetic mechanisms to preserve intracellular homeostasis. The epigenetic regulation of m^6^A in the autophagic process of human diseases has gradually come to light in recent studies [139]. In reviewing the literatures, considerable evidences were found on the association between m^6^A methylation and autophagy. The regulation of m^6^A methylation on autophagy can be negative or positive, which may be related to the different functions of modified RNAs during autophagy and the specific effect of m^6^A modification-related proteins on targeted RNAs.

## The direct regulatory role of m^6^A methylation in autophagy

The m^6^A modification directly regulates maturity of autophagy by affecting the activity of the autophagy-related proteins. One could argue that this regulation is negative. The stimulation of the ULK1 complex, which is comprised of ULK1 and the noncatalytic subunits FIP200 and ATG13, is the first step in the onset of autophagy. ALKBH5 maintained FIP200 at unmethylated steady-state levels, and YTHDF2 was unable to induce degradation of FIP200 in the cytoplasm because it failed to recognize m^6^A residues on the FIP200 transcript, resulting in increased FIP200 expression and activation of the autophagic pathway [140]. FTO-YTHDF2-dependent m^6^A methylation regulates the biological process of autophagy on ULK1 mRNA through the same mechanism as aforementioned [141]. Transcription factor EB (TFEB) is necessary for lysosomal biogenesis and autophagy [142, 143]. Increased m^6^A methylation of TFEB mRNA and decreased m^6^A expression level of TFEB mRNA are caused by upregulation of METTL3 and downregulation of ALKBH5 in ischemic heart disease, which together prevent the maturation of autophagy [144]. ATGs are a class of regulatory proteins that are essential for the formation of autophagosomes. Reduced FTO-mediated m^6^A modification on ATGs has been demonstrated to activate autophagy [145]. Moreover, when FTO is silenced, YTHDF2 binds to m^6^A methylation-enriched ATG5 and ATG7 transcripts, causing mRNA to decay and protein production to decline. This prevents the formation of autophagosomes [146]. Even though previous studies claimed that up-regulation of FTO would prevent autophagy from maturing [147]. The evidence presented thus far supports the idea that at the level of gene metabolism where m^6^A methylation regulates autophagy, the “writer” is primarily responsible for catalyzing m^6^A modification of RNA, while the “eraser” maintains the RNA in an unmethylated stable state, and it is the “reader” that ultimately determines the fate of the RNA. Furthermore, METTL3-mediated m^6^A modification reduces ATG7 expression by weakening the stability of ATG7 mRNA, the autophagic process is ultimately blocked [148]. Taken together, there are no studies on negative regulation factors of autophagy in this context, but the negativity of m^6^A methylation directly regulates autophagy is primarily reflected in the interaction between m^6^A modification-related proteins that can ultimately down-regulate the expression of autophagy-positive regulation factors.

However, such regulation may also be positive. YTHDF1 contributed to the translation of ATG2A and ATG14 by binding to the m^6^A site of methylated ATG2A and ATG14 mRNA, thus facilitating autophagy [149, 150]. Beclin1 contributes to the elongation of the autophagosome membrane. Several lines of evidence suggest that METTL14-YTHDF1-dependent m^6^A modification appears to trigger autophagy activation by stabilizing Beclin1 mRNA [151]. Consistently, METTL14-IGF2BPs-dependent m^6^A methylation plays the same role on Beclin1-mediated autophagy as above [152]. Moreover, down-regulated ALKBH5 promotes m^6^A methylation of Beclin1 and LC3 II/I mRNAs, resulting in the high expression of Beclin1 and LC3 II/I and activation of autophagy [153]. The presence of p62/SQSTM1-droplet, an autophagy selective receptor, creates a foundation for the formation of autophagosome [154], which may be related to recruiting more LC3. Therefore, the nuclear m^6^A methylation of SQSTM1 mRNA, which is mediated by YTHDC1, upregulates the expression of SQSTM1 and increases autophagic flux [76]. The evidences listed here suggest that the connection between proteins connected to m^6^A modification, which has the potential to up-regulate the expression of autophagy-positive regulation factors, is the main way that the positivity of m6A methylation directly regulates autophagy is manifested.

## The indirect regulatory role of m^6^A methylation in autophagy

The m^6^A modification can also indirectly regulate maturity of autophagy by affecting the activity of the autophagy-related pathways. Additionally, there are both negative and positive correlations between m^6^A alterations and pathways involved in autophagy. We preferentially focus on expanding the conversation around negative regulation. Autophagy is positively regulated by the AMPK pathway in the progression of sepsis-induced cardiomyopathy [155]. Previous research has shown that m^6^A methylation inhibits autophagy by increasing the translation of protein phosphatase 1A (PPM1A), an AMPK negatively regulated factor, which is mediated by YTHDF1, while decreasing the stability and expression of calcium/calmodulin-dependent protein kinase kinase 2 (CAMKK2), an AMPK actively regulated factor, which is mediated by YTHDF2 [156]. They also found that a decrease in METTL3 and METTL14 and an increase in ALKBH5 in the process [156]. In the same way, our research goes further and focuses on the LKB1, an upstream kinase of AMPK. WTAP-mediated m6A methylation impaired the stability and expression of LKB1 mRNA, which prevents the AMPK pathway from being activated and inhibits autophagic flux [157]. Additionally, SIRT1 pathway activates autophagy by deacetylating a variety of ATGs. Mechanically, METTL14-dependent m^6^A modification mediates the degradation of SIRT1 mRNA, which provide a potential possibility for curbing autophagy [158]. On the other hand, the synergy of tumor protein p53 inducible nuclear protein 2 (TP53INP2) with LC3 and ATG7 is also critical for autophagy activation. FTO induces the decreased m^6^A modification of TP53INP2 transcript as well as high expression of TP53INP2, which ultimately promotes autophagy [159]. In response to extracellular stress, the FOXO3 pathway maintains cellular homeostasis by acting on high levels of intracellular ROS to mediate autophagy [160]. METTL3-induced m^6^A methylation decreases autophagic flux through enhancing FOXO3 RNA stability and expression in an YTHDF1-dependent manner [161]. Similar to the above, Rubicon is a cellular autophagy negative regulator gene that binds to Beclin1 to inhibit the autophagic pathway; METTL3-YTHDF1-dependent m^6^A methylation also decreases autophagic flux by enhancing the stability and translation of Rubicon mRNA [162]. Initial observations suggest that PI3K/AKT/mTOR signaling pathway plays an important role in the anti-autophagy effect [163]. IGF2BP3 promotes translation machinery associated 7 homolog (TMA7) mRNA stability and translation through recognition of the m^6^A site on the TMA7 3’-UTR, which in turn activates the PI3K/AKT/mTOR pathway and ultimately inhibits autophagy [164]. Conversely, activating transcription factor 4 (ATF4) negatively regulates the mTOR signaling pathway. High expression of FTO maintained ATF4 mRNA at unmethylated steady-state levels, and YTHDF2 was unable to induce degradation of ATF4 in the cytoplasm because it failed to recognize m^6^A residues on ATF4 mRNA transcripts, thus increasing ATF4 expression levels and activating the mTOR-suppressed autophagic pathway [165]. Overall, the m^6^A modification negatively regulates autophagic activity mainly by mediating the expression of upstream signaling pathways of autophagy regulators.

Then, we develop the discussion of positive regulation. ALKBH5-mediated reduction of m^6^A methylation indirectly inhibits the development of autophagy by regulating the expression of GSK3β/mTOR signaling pathway [166]. Earlier, study has observed that miR-199a impairs autophagy in thick heart muscle cells in a cell-autonomous way through targeted GSK3β/mTOR signal pathway [167]. More precisely, autophagy is negatively regulated by the GSK3β/mTOR signal pathway [168]. USP13 is known to be an essential deubiquitinase that stabilizes ATG5 by deubiquitination. Mechanically, USP13’s m^6^A modification is catalyzed by METTL3, and IGF2BP2 promotes USP13 mRNA translation by identifying m^6^A residues on USP13 transcripts, ultimately triggering autophagy [169]. Decapping Protein 2 (DCP2) is degraded as a result of METTL3’s induction of m^6^A methylation, which facilitates mitophagy via the Pink1-Parkin pathway [170].

**Figure 3:**
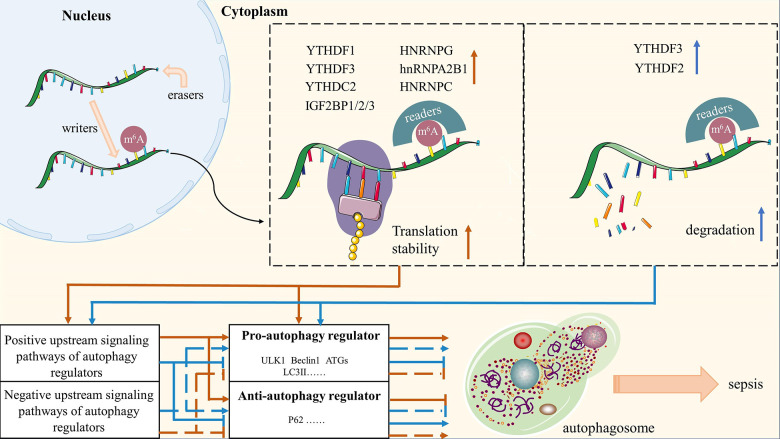
**A summary of molecular mechanisms of m^6^A-modified autophagy on sepsis.** Red arrows represent the role of m^6^A methylation in maintaining the stability of autophagy regulators and their upstream signaling pathways; Blue arrows represent the degradation of autophagy regulators and their upstream signaling pathways by m^6^A methylation.

To sum up, a sizable and intricate regulatory network of signaling pathways exists upstream of the autophagy regulators, and m^6^A modification in any member of this regulatory network will govern the bioprocess of autophagy by affecting the expression of its downstream signals. As shown in Figure 3, the aforementioned empirical findings in the present study provide a new understanding of m^6^A methylation regulates autophagy. First, we summarize the regulation of m^6^A methylation in the biological metabolism of autophagy as a “writer/eraser-reader-dependent” model, where the “writer” is mainly responsible for catalyzing the m^6^A modification of RNA, while the “eraser” maintains the RNA in an unmethylated state, and the “reader” is ultimately responsible for determining the fate of the RNA. Second, two approaches exist for m^6^A methylation to control autophagic activity: directly by altering the autophagic regulators themselves, or indirectly by altering the upstream signaling pathways that mediate the autophagic regulators’ expression. Then, m^6^A modification up-regulates autophagic activity by inhibiting the degradation of autophagy-negative factors or encouraging the expression of factors that promote autophagy. Conversely, it down-regulates autophagic activity. Last but not least, m^6^A modifications have an impact on autophagy regulators and autophagic activity by mediating the expression and degradation of the regulators’ upstream signaling pathways.

## The regulatory role of m^6^A methylation modified autophagy in sepsis

Autophagy is a conserved lysosomal degradation pathway that transports substrates (including large amounts of cytoplasm, organelles (e.g., mitochondria and peroxisomes), aggregation-prone proteins, and infectious agent) to lysosomes via double-membrane vesicles. The autophagy pathway plays homeostatic activities in protein and organelle quality control to maintain mammalian developmental and differentiation processes [171]. Although autophagy is generally regarded as an adaptable and protective biological process, it can be destroying when it occurs in excess or has defects. From the focus of studies on the biological functions of autophagy genes over the past two decades, autophagy specifically targets disease-causing proteins, intracellular microorganisms, and dysfunctional organelles; deficits in these processes that cause abnormal accumulations of inflammatory signals may be relevant to the pathophysiological mechanisms of inflammatory diseases [22]. Research shown that inhibiting autophagic flux increases the likelihood of non-canonical inflammasome pathways being activated, which impairs with the host’s ability to fight off infection [172]. Improved autophagic flux-based sepsis therapy options are suggested by a clinical investigation that reveals impaired autophagic flux in septic patients [173]. Our previous basic studies have revealed that activation of autophagy is protective of multiorgan function in a sepsis model [174, 175]. Then, cognitive dysfunction in sepsis-related encephalopathy is ameliorated by activation of PPAR-γ signaling pathway-mediated autophagy in astrocytes, as evidenced by high expression of LC3, ULK1, and low expression of P62 [176]. Conversely, inhibition of the autophagy pathway may likewise ameliorate sepsis-induced organic depression. In the pathogenesis of sepsis, for instance, it has become clear that suppressing autophagy mechanisms by targeting SIRT4, MAPKs, and Nrf2 pathways may be a useful strategy for protecting organ function [177–179]. Indeed, it has also been demonstrated that autophagy is activated early in the onset of sepsis, but that as the condition progresses, autophagic activity declines [180, 181]. There are opposing views in the existing literature on the crucial protective or destructive functions of autophagy in sepsis-induced organ damage. We speculate that the cause of this phenomenon might be connected to the respective alteration of post-transcriptional processes that autophagy regulators and their upstream signaling pathways go through during sepsis. Needless to say, the dynamic change of autophagy during the development of sepsis will continue to receive attention in the subsequent research. Consequently, the autophagy pathway appears to be closely involved in the pathogenesis of sepsis, and its modulation may be of therapeutic value in the clinical context. According to studies, the most common post-transcriptional modification is the m^6^A methylation, and the internal modifications it exerts in mRNAs are an intricate biological process [182]. Since the relationship between m^6^A methylation and autophagy has been elucidated in many human diseases [183], the molecular mechanisms by which m^6^A-autophagy interactions induce sepsis have received extensive attention from emergency physicians. The dual role of m^6^A modifications in sepsis is strikingly similar to that of autophagy, which can both promote and hinder the occurrence and development of sepsis [136, 184]. Furthermore, the mutual control of m^6^A modification and autophagy is becoming increasingly clear as a result of profound autophagy research, and their interactions can further affect the efficacy of sepsis therapy. Research on the sepsis-associated characteristic gene METTL3 has been increasingly prevalent in recent years, with the goal of exploring potential epigenetic treatment targets for sepsis patients. Under disease-related circumstances, the expression of METTL3 target genes can fluctuate, which affects the pathophysiology of inflammatory diseases by affecting the expression of downstream target genes [185]. A key pathogenic mechanism in sepsis, PINK1/Parkin-mediated mitochondrial autophagy, is negatively regulated by DCP2 [26]. The latest report indicates that the m^6^A methyltransferase METTL3 can facilitate mitochondrial autophagy mediated by the PINK1/Parkin pathway by triggering the m^6^A methylation of DCP2, which results in the degradation of DCP2 [170]. Additionally, it was discovered that METTL3-IGF2BP2-dependent m^6^A modification emerged as a contributing factor in the deterioration of sepsis-induced acute lung damage [184]. Likewise, such m^6^A modification means promote autophagy in certain diseases [186]. Further researches that METTL3-mediated m^6^A methylation inhibits the activation of autophagy also supported the anti-inflammatory function of this modulation in infectious illnesses [187]. More precisely, m^6^A modification leads to impaired autophagic flux ending in reduced cellular viability during sepsis-induced organ dysfunction [188]. Consequently, m^6^A methylation affects the regulation of autophagy during a dysregulated host response to infection in addition to being involved in the pathophysiology of sepsis and the development of autophagy. To summarize, m^6^A methylation modified autophagy may be the potential molecular mechanism and have clinical value in sepsis, but more research is needed.

## CONCLUSIONS

In this review, we present a hypothesis on “writer/eraser-reader-dependent” m^6^A methylation-modified autophagy that may aid in the discovery of novel therapeutic targets to reduce morbidity and mortality related to organ dysfunction subsequent to sepsis and give a theoretical basis for more comprehensive management of sepsis patients. Globally, the health of people is seriously threatened by the complex series of diseases known as sepsis. Over the last few decades, we have been working hard to uncover the underlying molecular mechanisms in sepsis. As sepsis progresses, there is growing evidence that alterations in gene expression and epigenetic regulation are related to organ dysfunction. It is well known that one of the recognized RNA modifications that controls epigenetic and gene expression is m^6^A methylation. In addition, a series of investigations have demonstrated that m^6^A methylation regulates a variety of biological processes in sepsis. On the other hand, m^6^A methylation plays a crucial role in the post-transcriptional steps of genes, affecting the stability, export, splicing and translation of the transcripts involved in the autophagic process. Previous studies have also confirmed the importance of maintaining the orderly and complete autophagic process in the prevention of sepsis. Unfortunately, there is only limited evidence elucidating a potential relationship between m^6^A-modified autophagy and sepsis. For example, a recent study revealed that METTL3 mediates the m^6^A methylation of SIRT1 mRNA, which suppresses SIRT1 protein expression and autophagic flux and eventually results in sepsis-induced acute lung injury [188]. In conclusion, the novel regulatory model of m^6^A methylation modification proposed in this paper provides an innovative research direction for the therapies of sepsis.

The following are some of the key points that needs to be covered in this article: (1) A gene’s whole post-transcriptional biological process is impacted by m^6^A methylation, and each of the proteins involved in this modification has a specific function in this process. By summarizing the current evidence on m^6^A modification-related proteins acting on the metabolism of RNA, we emphasize the importance of synergistic interactions among m^6^A modification-related proteins in regulating m^6^A methylation during the pathophysiology of disease. (2) We suggest a novel m^6^A modification model-“writer/eraser-reader-dependent” m^6^A methylation (Figure 2), and thoroughly analyze the specific molecular mechanisms by which it regulates sepsis. (3) At the level of gene metabolism where m^6^A methylation regulates autophagy, the “writer” is mainly responsible for catalyzing the m^6^A modification of RNA, while the “eraser” maintains the RNA in an unmethylated state, and the “reader” is ultimately responsible for determining the fate of the RNA. (4) In order provide new insights into the regulatory role of m^6^A modifications throughout the autophagic process, we have meticulously collated relevant potential mechanisms between m^6^A modifications and autophagy, including direct evidence for particular molecular mechanisms and indirect evidence for pertinent signaling pathways (Figure 3). (5) We speculate that the pathophysiology of sepsis may also be influenced by the putative molecular mechanisms between m^6^A modification and autophagy. However, there is still no clear evidence for the effect of m^6^A-modified autophagy on sepsis, and further exploration of potential links between the listed mechanisms is required.

## Supplementary Material

Supplementary Table 1
