# Supplementary material for: Novel insights into the regulatory role of N6-methyladenosine methylation modified autophagy in sepsis
Source: Aging (Albany NY). 2023 Dec 18;15(24):15676–700. doi: 10.18632/aging.205312 (PMC10781468; doi:10.18632/aging.205312)
Supplement: Supplementary Table 1 [file aging-15-205312-s001.docx]

Supplementary Table 1. Effects of m6A on RNA metabolism.

| m^6^A methylation modification-related proteins | | Modified RNAs | Diseases | Findings | Refs |
| --- | --- | --- | --- | --- | --- |
| writers | METTL3 | PKC-η mRNA | diabetes | PKC-η, FAT4, and PDGFRA mRNAs expression rose as a result of the reduction of m^6^A methylation brought on by METTL3 silencing, which prevented diabetes-related peripapillary cell dysfunction and reduced retinal vascular complications. The protective effect of METTL3 was, however, abrogated by overexpression of YTHDF2 | [43] |
|  |  | FAT4 mRNA |  |  |  |
|  |  | PDGFRA mRNA |  |  |  |
|  |  | SLC7A11 m6A | hepatoblastoma | IGF2BP1 recognizes METTL3-mediated m^6^A modification of SLC7A11 mRNA and prevents the recruitment of the BTG2/CCR4-NOT complex to PABPC1 by competitive binding to PABPC1 and inhibits the deacetylation of SLC7A11 mRNA, ultimately leading to tumorigenesis in hepatoblastoma. | [44] |
|  |  | LncRNA D63785 | neuronal cell injury | Through enhancing METTL3-dependent Lnc-D63785 m^6^A methylation, oxygen glucose deprivation/re-oxygenation (OGD/R) decreases Lnc-D63785 expression, causing an accumulation of miR-422a and the apoptosis of neuronal cells. | [45] |
|  |  | FBXW7 mRNA | lung adenocarcinoma (LUAD) | By embedding m^6^A modification in the CDS of FBWX7 mRNA, METTL3 regulates FBXW7's potential to act as a tumor suppressor, which eventually promotes apoptosis and inhibits cell proliferation via FBXW7 to decrease growth in LUAD. | [46] |
|  |  | ZMYM1 mRNA | gastric cancer | The HuR (known as m^6^A binding protein) binding site on ZMYM1 was targeted by METTL3-mediated m^6^A methylation modification to increase ZMYM1 expression in the genesis of gastric cancer. | [47] |
|  |  | miR-143 | myocardial infarction | METTL3 enhanced the m^6^A modification of pri-miR-143, which resulted in the transition of pri-miR-143 into mature miR-143-3p; miR-143-3p significantly reduce the expression of Yap and Ctnnd1; miR-143-3p blocked cardiomyocytes proliferation via its interaction with Yap and Ctnnd1. | [48] |
|  |  | lncRNA H19 |  | Protective effect of hypoxic preconditioning (HPC) was dependent on METTL3/METTL14-mediated abundant m^6^A methylation and overexpression of lncRNA H19. | [49] |
|  | METTL14 |  |  |  |  |
|  |  | miR-149-3p | intestinal  inflammation and Colorectal carcinoma (CRC) | MiR-149-3p was down-regulated by enterotoxigenic bacteroides fragilis, leading to the development of CRC and intestinal inflammation. This process was controlled by METTL14-dependent m^6^A methylation, which regulated the processing of pri-miR149 by DGCR8. | [50] |
|  |  | SOX4 mRNA | CRC | METTL14 mediates m^6^A methylation of SOX4 mRNA, reduces SOX4 mRNA expression and inhibits the deterioration of CRC | [51] |
|  |  | lncRNA XIST |  | Knockdown of METTL14 induces the low m^6^A methylation level of lncRNA XIST, augments lncRNA XIST expression and promotes tumorigenicity and metastasis of CRC | [52] |
|  |  | CYP1B1 | cervical carcinoma | METTL14 is influenced by its upstream signal (piRNA-14633) to induce the m^6^A methylation of CYP1B1 and promote the malignancy of cervical carcinoma. | [53] |
|  |  | lncRNA TINCR | diabetes | The expression of the lncRNA TINCR was inhibited by METTL14-mediated m^6^A methylation modification, which lowered the stability of the NLRP3 mRNA and caused its downregulation. Finally, downregulation of NLRP3 prevented the progression of diabetes cardiomyopathy. | [54] |
|  | METTL16 | lncRNA RAB11B-AS1 | hepatocellular carcinoma | lncRNA RAB11B-AS1 was directly bound to by METLL16, which also caused lncRNA RAB11B-AS1 to undergo m^6^A modification, lowered lncRNA RAB11B-AS1 transcript stability, and thus downregulated lncRNA RAB11B-AS1 transcript level. Eventually, the tumor-suppressive roles of RAB11B-AS1 in hepatocellular carcinoma reversed. | [55] |
|  |  | cyclin D1 mRNA | gastric cancer | METTL16 enhances the stability and expression of cyclin D1 mRNA in gastric cancer cells through m^6^A modification. Subsequently, this accelerates gastric cancer cells cycle. | [56] |
|  | WTAP | MXD2 mRNA | mTORC1-driven cancers | mTORC1 increases m^6^A methylation on MXD2 mRNA promotes its degradation by increasing the expression of methyltransferase complex regulatory subunit, WTAP, through eIF4A/4B-dependent translation. | [57] |
|  |  | ETS1 mRNA | hepatocellular carcinoma | The WTAP-regulated modification of m^6^A led to post-transcriptional repression of ETS1, which resulted in the down-regulation of ETS1 mRNA expression that would have prevented the development of hepatocellular carcinoma, eventually enhancing the proliferative potential and tumor growth of hepatocellular carcinoma cells. | [58] |
|  | KIAA1429(VIRMA) | GATA3 mRNA |  | KIAA1429 promoted m^6^A methylation on the 3' UTR of GATA3 pre-mRNA, subsequently causing the dissociation of the RNA-binding protein HuR and the degradation of GATA3 pre-mRNA | [59] |
|  |  | SIRT1 mRNA | CRC | KIAA1429 promotes colorectal tumor growth via regulating the expression and stability of SIRT1 mRNA in an m^6^A-dependent manner. | [60] |
|  |  | BTG2 mRNA | lung adenocarcinoma | The m^6^A levels of BTG2 mRNA was reduced by the knockdown of KIAA1429, which also contributed to the carcinogenesis of lung adenocarcinoma by stimulating the expression of BTG2 and increasing the YTHDF2-dependent stability of BTG2 mRNA. | [61] |
|  |  | lncRNA CCAT1 | prostate cancer | By generally lowering m6A levels and lowering the stability and abundance of carcinogenic lncRNAs CCAT1 and CCAT2, VIRMA downregulation reduces the aggressive phenotype of prostate cancer. | [62] |
|  |  | lncRNA CCAT2 |  |  |  |
|  | RBM15 | TMBIM6 mRNA | Laryngeal squamous cell cancer (LSCC) | RBM15 enhances the m^6^A modification of TMBIM6 mRNA; subsequently, IGF2BP3 bound to the m^6^A site in the 3’UTR region of TMBIM6 and strengthen the stability of TMBIM6. Ultimately, this progress promotes the proliferation of LSCC. | [63] |
|  |  | HK2 mRNA | osteosarcoma | The interaction between circ-CTNNB1 and RBM15 led to the promotion of HK2, GPI, and PGK1 expression through m^6^A modification, which in turn facilitated in the glycolysis process and triggered osteosarcoma development. | [64] |
|  |  | GPI mRNA |  |  |  |
|  |  | PGK1 mRNA |  |  |  |
|  |  | CLDN4 mRNA | Gestational diabetes Mellitus | RBM15 emerges as insulin resistance by directing the m^6^A modification of CLDN4 and decreasing CLDN4 expression. | [65] |
| erasers | ALKBH5 | Drp1 mRNA | liver fibrosis | Loss of ALKBH5 enhances the m^6^A modification of Drp1 mRNA; YTHDF1 discerns the above m^6^A site and increases Drp1 mRNA stability, which promotes mitochondrial fission, eventually leading to hepatic stellate cell proliferation and migration and liver fibrosis. | [66] |
|  |  | SOCS3 mRNA | osteosarcoma | ALKBH5 reduces the m^6^A modification of SOCS3 mRNA, which prevents YTHDF2-mediated SOCS3 degradation and increases SOCS3 expression. This inactivates the STAT3 pathway, which blocks osteosarcoma proliferation and growth. | [67] |
|  |  | WIF-1 mRNA | pancreatic cancer | ALKBH5 reduces the m^6^A modification of WIF-1 mRNA and upregulates the expression of WIF-1 protein, which inactivates the Wnt pathway and inhibits pancreatic tumorigenesis. | [68] |
|  |  | CCL28 mRNA | acute kidney injury (AKI) | By boosting CCL28 m^6^A methylation to upregulate CCL28 levels, ALKBH5 deletion enhances CCL28 mRNA stability. Increased CCL28 levels attract Treg cells, which shield the kidney from being inhibited by the invasion of inflammatory cells. | [69] |
|  | FTO |  | heart failure; myocardial ischemia | In failing hearts, FTO expression reduces, which causes an abnormal rise in transcriptome-wide m^6^A methylation and a decline in the contractile activity of the cardiomyocytes. | [36, 70] |
|  |  | FOS mRNA | ovarian aging | The m^6^A methylation of abundant FOS mRNA in the 3′UTR is brought about by FTO downregulation, and IGF2BP2 recognizes the m^6^A locus on FOS and supports the stability and translation of mRNA, ultimately causing ovarian aging. | [71] |
| readers | YTHDF1 | CNOT7 mRNA | osteosarcoma | Osteosarcoma cells have increased YTHDF1 expression, which stimulates recognition of CONT7 translation initiation by recognizing the m^6^A region of CONT7 to encourage cell proliferation, migration, and invasion. | [72] |
|  | YTHDF2 | LHPP mRNA | prostate cancer | By identifying the m^6^A methylation sites on cancer suppressor genes (LHPP and NKX3–1), YTHDF2 triggers mRNA degradation, which drives prostate cancer proliferation and migration. | [73] |
|  |  | NKX3–1 mRNA |  |  |  |
|  |  | UBXN1 mRNA | glioma | By identifying the m^6^A methylation sites on UBXN1 mRNA, YTHDF2 triggers mRNA degradation, which actives NF-κB pathway and drives glioma proliferation and migration. | [74] |
|  |  | Myh7 mRNA | cardiac hypertrophy | YTHDF2 suppresses cardiac hypertrophy via recognizing the m^6^A site on Myh7 mRNA to promote its degradation | [75] |
|  |  | PKC-η mRNA | diabetes | PKC-η, FAT4, and PDGFRA mRNAs expression rose as a result of the reduction of m^6^A methylation brought on by METTL3 silencing, which prevented diabetes-related peripapillary cell dysfunction and reduced retinal vascular complications. The protective effect of METTL3 was, however, abrogated by overexpression of YTHDF2 | [43] |
|  |  | FAT4 mRNA |  |  |  |
|  |  | PDGFRA mRNA |  |  |  |
|  | YTHDC1 | SQSTM1 mRNA |  | In the pathophysiology of diabetes, downregulation of YTHDC1 inhibits the initiation of SQSTM1 m^6^A methylation, which results in lower levels of SQSTM1 expression. This impairs autophagic flux and keratinocyte migration, which ultimately delays the healing of wounds. | [76] |
|  | IGF2BP1 | SLC7A11 m6A | hepatoblastoma | IGF2BP1 recognizes METTL3-mediated m^6^A modification of SLC7A11 mRNA and prevents the recruitment of the BTG2/CCR4-NOT complex to PABPC1 by competitive binding to PABPC1 and inhibits the deacetylation of SLC7A11 mRNA, ultimately leading to tumorigenesis in hepatoblastoma. | [44] |
|  | IGF2BP2 | slug mRNA | head and neck squamous carcinoma (HNSCC) | IGF2BP2 detects the m^6^A site in the coding sequence (CDS) region of slug and binds to it, increasing the stability of the mRNA. Slug, which is highly expressed, initiates the epithelial-mesenchymal transition and encourages the invasion and migration of HNSCC cells. | [77] |
|  |  | TAB3 mRNA | AKI | METTL3 mediates the m^6^A methylation of TAB3 mRNA. IGF2BP2 improves TAB3's stability by combining the m^6^A site on termination codec region of TAB3, which induces the inflammatory effect of TAB3. | [78] |
|  |  | TIMP2 mRNA | diabetic nephropathy | METTL3 mediates the m^6^A methylation of TIMP2 mRNA. IGF2BP2 recognizes the m^6^A site on TIMP2 and improves TAB3's stability, which regulates Notch signaling and induces podocyte injury (inflammation and apoptosis) in diabetic nephropathy. | [79] |

Abbreviations: SLC7A11 (Solute carrier family 7 member 11); FBXW7 (F-box and WD repeat domain-containing 7); ZMYM1 (zinc finger MYM-type containing 1); SOX4 (SRY-related high-mobility-group box 4); TINCR (terminal differentiation-induced non-coding RNA); MXD2 (MAX dimerization protein 2); ETS1 (ETS proto-oncogene 1); mTORC1 (mechanistic target of rapamycin complex 1); GATA3 (GATA Binding Protein 3); SIRT1 (Silencing information regulator 1); CCAT1 (colon cancer associated transcript 1); CCAT2 (colon cancer associated transcript 2); TMBIM6 (transmembrane BAX inhibitor motif containing 6); HK2 (hexokinase 2); GPI (glucose-6-phosphate isomerase); PGK1 (phosphoglycerate kinase 1); SOCS3 (Suppressor Of Cytokine Signaling 3); WIF-1 (Wnt inhibitory factor 1); CNOT7 (CCR4-NOT transcription complex subunit 7); UBXN1 (UBX domain protein 1); Myh7 (beta-myosin heavy chain); SQSTM1 (sequestosome 1); TAB3 [TGF-β-activated kinase 1 (MAP3K7) binding protein 3]; TIMP2 (TIMP Metallopeptidase Inhibitor 2).
